# Burden of influenza‐associated outpatient influenza‐like illness consultations in China, 2006‐2015: A population‐based study

**DOI:** 10.1111/irv.12711

**Published:** 2019-12-23

**Authors:** Luzhao Feng, Shuo Feng, Tao Chen, Juan Yang, Yiu Chung Lau, Zhibin Peng, Li Li, Xiling Wang, Jessica Y. T. Wong, Ying Qin, Helen S. Bond, Juanjuan Zhang, Vicky J. Fang, Jiandong Zheng, Jing Yang, Peng Wu, Hui Jiang, Yangni He, Benjamin J. Cowling, Hongjie Yu, Yuelong Shu, Eric H. Y. Lau

**Affiliations:** ^1^ Key Laboratory of Surveillance and Early‐warning on Infectious Disease Division of Infectious Disease Chinese Center for Disease Control and Prevention Beijing China; ^2^ WHO Collaborating Centre for Infectious Disease Epidemiology and Control School of Public Health Li Ka Shing Faculty of Medicine The University of Hong Kong Hong Kong Special Administrative Region China; ^3^ National Institute for Viral Disease Control and Prevention Collaboration Innovation Center for Diagnosis and Treatment of Infectious Diseases Chinese Center for Disease Control and Prevention Beijing China; ^4^ Key Laboratory of Public Health Safety Ministry of Education School of Public Health Fudan University Shanghai China; ^5^ School of Public Health (Shenzhen) Sun Yat‐sen University Shenzhen China

**Keywords:** disease burden, influenza, influenza‐like illness, surveillance, vaccination

## Abstract

**Background:**

Human influenza virus infections cause a considerable burden of morbidity and mortality worldwide each year. Understanding regional influenza‐associated outpatient burden is crucial for formulating control strategies against influenza viruses.

**Methods:**

We extracted the national sentinel surveillance data on outpatient visits due to influenza‐like‐illness (ILI) and virological confirmation of sentinel specimens from 30 provinces of China from 2006 to 2015. Generalized additive regression models were fitted to estimate influenza‐associated excess ILI outpatient burden for each individual province, accounting for seasonal baselines and meteorological factors.

**Results:**

Influenza was associated with an average of 2.5 excess ILI consultations per 1000 person‐years (py) in 30 provinces of China each year from 2006 to 2015. Influenza A(H1N1)pdm09 led to a higher number of influenza‐associated ILI consultations in 2009 across all provinces compared with other years. The excess ILI burden was 4.5 per 1000 py among children aged below 15 years old, substantially higher than that in adults.

**Conclusions:**

Human influenza viruses caused considerable impact on population morbidity, with a consequent healthcare and economic burden. This study provided the evidence for planning of vaccination programs in China and a framework to estimate burden of influenza‐associated outpatient consultations.

## 1 INTRODUCTION

Pandemic and seasonal influenza viruses cause substantial morbidity and mortality worldwide and in China.^1^, ^2^, ^3^, ^4^, ^5^ The viruses are associated with a large number of excess deaths, hospitalizations, and outpatient visits as well as absences from work or school resulting from infections. Population‐based burden estimation found persons of all ages were associated with substantial burden of outpatient visits and associated absenteeism. In the United States, the Influenza Incidence Surveillance Project reported a national estimate of around 10 per 1000 persons consulted because of influenza A(H1N1) in 2009/10 pandemic season, of which the highest age‐specific rates occurred among children aged below 17 years.^6^

Currently, population‐based estimates of influenza‐associated excess deaths and hospitalizations have been increasingly reported,^2^, ^5^, ^7^, ^8^ while few studies have been published on influenza‐associated outpatient visits which may also bring considerable economic burden to the society.^3^, ^6^, ^9^, ^10^, ^11^ These studies were mostly limited to a single season or region (state/province/city/county), for a specific age group, or reported overall rather than region‐specific estimates.^3^, ^9^, ^10^ Meanwhile, few evidence on influenza‐associated outpatient burden among Chinese population is available.^10^, ^12^, ^13^ The challenges in estimating population‐level influenza‐associated outpatient burden may arise from the difficulty in distinguishing symptoms caused by influenza viruses from those by other respiratory virus (eg, RSV or rhinovirus). Compared with other clinical case definitions, the commonly used definition of influenza‐like illness (ILI) is relatively broad to capture influenza‐associated illness. Another challenge lies in determining the underlying population covered by surveillance networks. Studies from the United States or UK used the number of population registered with general practitioners as the underlying population; however, such data may not be available in developing countries, for example, China.

Influenza seasonality varies across countries and may exhibit different patterns within countries covering a wide range of latitudes.^14^ Understanding regional influenza‐associated ILI burden plays a key role in formulating and evaluating national control strategy for seasonal influenza. To determine province‐specific and age‐specific influenza‐associated outpatient burden, and further guide influenza vaccination recommendations, we estimate the influenza‐associated ILI outpatient burden in 30 provinces in China from 2006 to 2015 including the pandemic year 2009.

## 2 METHODS

### 2.1 Influenza surveillance and virological data

The national sentinel hospital‐based ILI surveillance system was initiated by the Ministry of Health in 2000, in line with the recommendations by the World Health Organization.^15^ Since October 2005, the system has incorporated 193 sentinel surveillance hospitals from 30 provinces, among which 12 of the 15 northern provinces conducted winter surveillance and all 15 southern provinces conducted surveillance throughout the year. After the 2009 influenza pandemic, the system was expanded to 554 sentinel hospitals with all provinces implementing year‐round surveillance. The surveillance departments included pediatrics (both outpatient and A&E, targeting patients below 15 years old), general medicine (both outpatient and A&E, targeting patients aged 15 years or above) and fever clinics (separated areas for screening suspected patients with notifiable infectious diseases).

Sentinel hospitals recorded weekly age‐specific number of outpatient consultations and number of outpatient consultations with ILI symptoms, defined as fever (≥38°C) plus cough or sore throat. Nasopharyngeal swabs were taken from first one or two cases presented with ILI each day in the sentinel hospitals and sent to city or province‐level network laboratory. The weekly number of samples collected from each sentinel hospital mostly ranged from 10 to 15 during influenza seasons. Network laboratories utilized Madin‐Darby canine kidney cells and/or chicken embryo isolating influenza viruses. Viruses were further typed/subtyped by hemagglutination inhibition and/or real‐time reverse transcription polymerase chain reaction (RT‐PCR) assay (initiated in May 2009). To account for the different sensitivity to influenza detection between RT‐PCR and virus culture, we adjusted specimens that were tested positive by RT‐PCR using an adjustment ratio based on a previous study.^5^ The laboratories used the protocols and test kits released by the Chinese National Influenza Centre, a World Health Organization Collaborating Centre for Reference and Research on Influenza. The national surveillance system has been described elsewhere in detail.^2^, ^14^ The same surveillance protocol and ILI definition were used in all sentinel hospitals. As public health surveillance data and routinely collected specimens were used, according to the National Health Commission of China national influenza surveillance guidelines, this project was considered official public health surveillance activities and institutional review board approval was not required.^16^ In our study, we retrieved data from the national surveillance system in China from 2006 to 2015. We performed data cleaning and weighting to improve data validity and representativeness (Appendix S1).

### 2.2 Meteorological, geographic, and socioeconomic data

Daily and city‐level temperature and relative humidity from 2006 through 2015 were obtained from the China Meteorological Administration. Province‐level meteorological data were aggregated as the mean of city‐level data and then averaged into weekly‐level. Missing meteorological data (0.07%) were imputed by averaging the weekly data in the same province from other years.

We obtained data on administrative divisions and areas from the State Council, the People's Republic of China,^17^ and age‐specific annual population size from National Bureau of Statistics of China.^18^ Annual number of outpatient (medicine and pediatrics) were obtained for each province from 2007 to 2014.^18^ Missing data in 2015 and 2016 (20%) from annual provincial on population and outpatients were imputed by data from other years using linear regressions using year as predictor. Weekly number of the population and outpatients were interpolated from annual data by natural spline method.

To further explore potential socioeconomic factors associated with disease burden, we also collected data for each province on total and urban area in 2015, per capita gross regional product (perGRP), per capita disposable income (perDI), number of hospitals, number of beds in hospitals, number of outpatient departments in 2015, per capita annual consumption expenditure of urban households on health care and medical services (perUHCMS), and per capita annual consumption expenditure of rural households on health care and medical services (perRHCMS) in 2012 from the National Bureau of Statistics of China.^18^

### 2.3 Statistical methods

We assumed the proportion of ILI consultation among medicine and pediatric outpatient consultations in the surveillance hospitals is representative of the province. Hence, the weekly total influenza‐associated ILI burden was calculated as the product of weekly total influenza‐associated ILI consultation proportion (among surveillance departments including medicine and pediatrics) in surveillance hospitals and outpatient consultation proportion in the population.

$$\begin{array}{c} \frac{\text{Influenza-associated ILI consultations in a province}}{\text{Total population in a province}}\\ =\frac{\text{Total outpatient consultations in a province}}{\text{Total population in a province}}\\ \times \frac{\text{Total influenza-associated ILI consultations in surveillance hospitals}}{\text{Total outpatient consultations in surveillance hospitals}}. \end{array}$$

A wide variety of pathogens may lead to ILI medical consultations. Here, we adopted a well‐established strategy by first linking the observed ILI medical consultations to influenza virus activity in a statistical model, and then, the influenza‐associated ILI burden was estimated by quantifying the excess ILI consultations due to influenza virus.^19^ Generalized additive regression models were used to estimate weekly influenza‐associated ILI burden, accounting for the time‐varying baseline and potential non‐linear effects.^8^, ^20^ We assumed an additive association between influenza‐associated ILI burden and influenza virus detection rate, and thus, linear model with an identity link was used.^20^ Weekly province‐specific virus detection rates of influenza A (H1N1), A(H3N2), A(H1N1pdm) and influenza B were predictors in the model. We also included potential confounding factors such as temperature and absolute humidity in the models,^21^ and spline terms to allow for potential non‐linear relations.^8^ Dummy variable was used to allow for potential instantaneous effect due to changes in reporting and laboratory methods during influenza pandemic period from May 11, 2009, to January 17, 2010.^22^ In the sensitivity analysis, we assumed that the above change would result in a long term effect and we also allowed for potential change due to expansion of the surveillance network after the pandemic. Further information on main and sensitivity analysis were described in Appendix S1.

We anticipated influenza‐associated ILI burden may vary across provinces. To understand factors behind, we tested several socioeconomic factors which could be associated with population health‐seeking behavior using a cluster analysis (further details in Appendix S1).^5^ Analyses were conducted using R version 3.3.3.

## 3 RESULTS

### 3.1 Socioeconomic status and healthcare resources

The national influenza surveillance system covered 30 provinces, and the surveillance hospitals provided an average of 3.1% (5.5% in 2009) of all medical consultations in the study period. Highest per capita GRP was observed in Tianjin, Beijing, and Shanghai municipalities, and Jiangsu and Zhejiang provinces (Table S1). Healthcare resources, indicated by number of hospitals, number of hospital beds, and number of outpatient department, varied by provinces (Table S1). We examined correlations between healthcare resources variables and socioeconomic variables across provinces, and found strong associations between number of hospital/number of outpatient department (ρ = 0.802, *P* < .001) and population (ρ = 0.797, *P* < .001) only. No strong correlation was observed between healthcare resources and income or expenditure.

### 3.2 ILI consultation and virological testing

During the 10‐year period from 2006 to 2015, a total of 568 sentinel hospitals participated in the national influenza surveillance in China, comprising 5293 hospital‐year. After the data cleaning procedures, we excluded 546 (10.3%) hospital‐year from further analysis (Figures S1 and S2).

The pediatrics and medical departments of sentinel surveillance hospitals provided a total of 785 720 000 medical consultations over 10 years, among which 2 540 000 (3.1%) were ILI consultations during 2006‐2015 (excl. 2009), and were 4.7% and 2.9% in the pre‐pandemic and post‐pandemic period, respectively. This proportion peaked at 5.5% in 2009. This pattern was also noticeable in all provinces except Jilin. However, ILI consultation rates varied substantially across provinces. For example, the annual ILI consultation rate was highest in Tianjin municipality (8.1% in 2006‐2015 excl. 2009), and lowest in Ningxia, Qinghai, and Chongqing (0.5%, 1.3% and 1.0% respectively, in 2006‐2015 excl. 2009) (Table S1). On average, 231 276 specimens (representing 9.1% of ILI medical consultations) were sent to the network laboratories each year from 2006 to 2015 excluding 2009, among which 10% were detected positive for influenza. In 2009, influenza‐positive rate almost doubled that in other years (19.6%) though similar numbers of specimens (250 411) were collected by sentinel hospitals (Table S1). Mean annual specimen tested and annual influenza‐positive rate were also varied by provinces: Beijing, Shanghai, and Tianjin municipalities detected highest influenza‐positive rates (Table S1).

The heatmaps in Figures S3 and S4 demonstrated weekly ILI consultation rate and laboratory‐confirmed influenza‐positive rate from 2006 to 2015, by province ordered in descending latitude. Before 2009, seasonal pattern was less clear particularly in the southern provinces. However, after the pandemic year 2009, provinces from the north demonstrated clearer annual influenza winter peak, while southern provinces presented another peak annually during summer (Figures S3 and S4). Provinces in central China had mixed pattern. The pattern of ILI consultation rate was less clear between provinces but generally consistent with virological activity.

### 3.3 Influenza‐associated ILI burden

**Figure 1:**
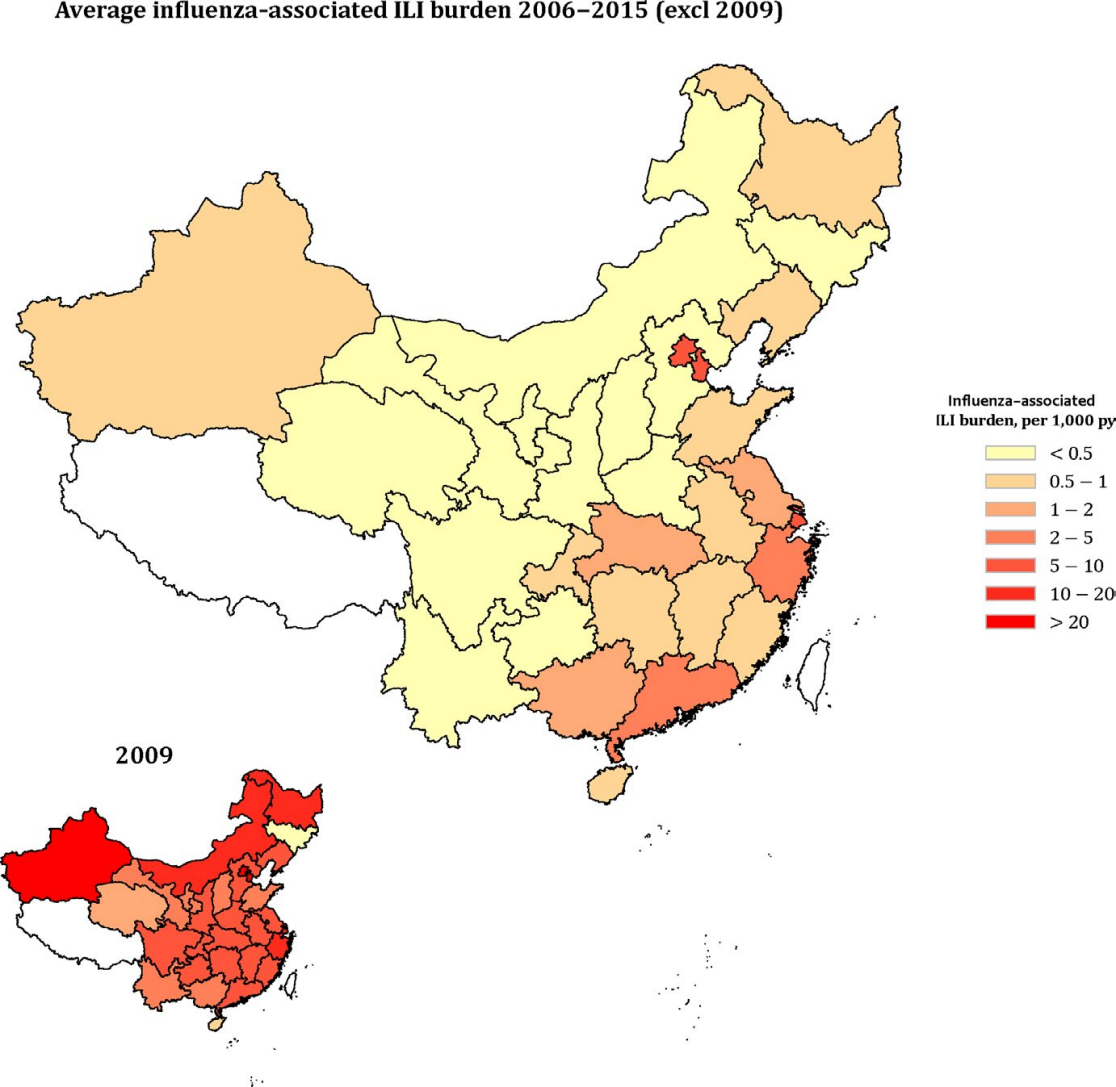
Average influenza‐associated ILI burden in 30 provinces in China, 2006‐2015 exclude 2009 and in 2009

Generalized linear additive models showed good fit across all 30 provinces, explaining 63.5%‐93.8% of the variation in observed influenza‐associated ILI consultations (Figure S5). Overall, influenza viruses were associated with 2.5 (95% CI: 1.5, 3.6) consultations per 1000 person‐years (py) in China each year from 2006 to 2015 (Table 1). The pandemic in 2009 led to higher influenza‐associated consultations across all provinces compared to other years, with a national mean of 7.8 (95% CI: 6.1, 9.6) consultations per 1000 py (Table 1). After the pandemic year, the highest influenza‐associated ILI burden was observed in 2014 when seasonal influenza activity was most prominent (Figure S4), with 1.9 (95% CI: 1.1, 2.9) consultations per 1000 py, and lowest was observed in 2011, with 1.0 (95% CI: 0.4, 1.6) per 1000 py consulted attributable to influenza infection. Large variations of influenza‐associated ILI burden were observed among 30 provinces (Table 1, Figure 1). Highest influenza‐associated ILI burden was estimated in Beijing, Tianjin, and Shanghai municipalities, ranging from 6.2 to 8.9 per 1000 py, while lowest burden in Jilin, Ningxia, and Qinghai provinces (Table 1, Figure 1). The sensitivity analysis shows similar estimates in influenza‐associated ILI burden (Table S2).

**Table 1:** Influenza‐associated influenza‐like illness consultations in 30 provinces in China, 2006‐2015 (per 1000 populations)

	2006	2007	2008	2009	2010	2011	2012	2013	2014	2015	Mean
Heilongjiang	0.6 (0, 1.3)	0.7 (0.1, 1.2)	0.4 (0.0, 0.8)	11.5 (10.2, 13.1)	0.6 (0.0, 1.4)	0.3 (0.0, 0.8)	0.4 (−0.5, 1.6)	0.8 (0.1, 1.7)	1.3 (0.2, 2.8)	0.7 (0.0, 1.6)	1.7 (1.0, 2.6)
Xinjiang	0.6 (0.1, 1.1)	0.2 (−0.1, 0.5)	0.4 (0.1, 0.9)	25.6 (22.2, 28.2)	1.1 (0.7, 1.6)	0.7 (0.3, 1.2)	0.9 (0.4, 1.6)	1.5 (0.7, 2.5)	1.9 (0.9, 3.2)	0.6 (0.2, 1.0)	3.3 (2.5, 4.2)
Inner Mongolia	0.1 (0.0, 0.1)	0.7 (0.1, 1.5)	0.4 (0.2, 0.7)	11.9 (9.5, 14.7)	0.6 (0.0, 1.2)	0.1 (−0.2, 0.5)	0.6 (−0.2, 1.6)	0.4 (−0.3, 1.2)	0.6 (−0.2, 1.5)	0.3 (−0.2, 0.9)	1.6 (0.9, 2.4)
Liaoning	0.7 (0.4, 1.0)	1.5 (1.1, 1.9)	0.5 (0.3, 0.6)	9.7 (9.0, 10.5)	0.8 (0.6, 1.0)	0.3 (0.2, 0.4)	0.8 (0.6, 1.1)	1.1 (0.8, 1.5)	1.6 (1.1, 2.0)	0.8 (0.6, 1.0)	1.8 (1.5, 2.1)
Jilin	0.2 (0.0, 0.3)	0.2 (−0.1, 0.6)	0.4 (0.0, 0.7)	‐0.9 (−1.5, −0.2)	0.1 (−0.2, 0.5)	0.2 (0.0, 0.4)	0.1 (−0.1, 0.4)	0.2 (−0.1, 0.4)	0.2 (−0.1, 0.6)	0.0 (−0.1, 0.2)	0.1 (−0.2, 0.4)
Beijing	3.9 (3.0, 4.9)	4.3 (3.3, 5.4)	3.0 (1.8, 4.2)	27.6 (23.4, 31.1)	9.2 (7.5, 11.0)	3.8 (3.1, 4.4)	7.2 (5.9, 8.6)	9.1 (7.6, 10.5)	14.9 (12.5, 17.2)	6.2 (4.6, 7.8)	8.9 (7.3, 10.5)
Tianjin	5.5 (3.9, 7.1)	7.2 (5.1, 9.4)	8.5 (6.2, 10.8)	23.5 (18.7, 28.2)	6.9 (5.1, 8.8)	5.0 (3.6, 6.3)	9.6 (6.7, 12.7)	6.1 (4.4, 7.7)	8.6 (6.6, 10.7)	4.7 (3.3, 6.3)	8.6 (6.5, 10.8)
Hebei	0.2 (0.0, 0.4)	0.3 (0.1, 0.6)	0.2 (0.0, 0.3)	6.1 (5.4, 6.8)	0.5 (0.3, 0.7)	0.3 (0.2, 0.5)	0.7 (0.3, 1.1)	0.6 (0.4, 0.9)	0.8 (0.5, 1.2)	0.3 (0.1, 0.5)	1.0 (0.7, 1.3)
Ningxia	0.0 (−0.1, 0.1)	0.0 (−0.1, 0.1)	0.1 (−0.4, 0.5)	2.3 (1.8, 3.1)	0.2 (0.0, 0.4)	0.2 (−0.1, 0.4)	0.1 (−0.4, 0.5)	0.2 (0.0, 0.5)	0.1 (−0.2, 0.4)	0.0 (−0.2, 0.1)	0.3 (0.0, 0.6)
Shanxi	0.0 (0.0, 0.1)	0.2 (0.1, 0.4)	0.1 (−0.2, 0.5)	3.4 (2.8, 3.9)	0.8 (0.4, 1.1)	0.4 (0.3, 0.6)	0.8 (0.4, 1.2)	0.5 (0.3, 0.7)	0.7 (0.4, 1.0)	0.2 (0.1, 0.3)	0.7 (0.5, 1.0)
Gansu	0.1 (−0.1, 0.2)	0.0 (−0.2, 0.3)	0.1 (0.0, 0.2)	3.0 (2.5, 3.6)	0.2 (0.0, 0.4)	0.0 (0.0, 0.1)	0.1 (−0.1, 0.3)	0.2 (0.0, 0.3)	0.2 (0.0, 0.4)	0.0 (0.0, 0.1)	0.4 (0.2, 0.6)
Shandong	0.1 (0.0, 0.3)	1.2 (0.9, 1.6)	0.6 (0.4, 0.9)	3.0 (2.4, 3.7)	1.0 (0.7, 1.4)	0.7 (0.5, 1.0)	0.8 (0.5, 1.0)	1.0 (0.7, 1.3)	1.0 (0.8, 1.3)	0.7 (0.5, 0.9)	1.0 (0.7, 1.3)
Qinghai	0.1 (0.0, 0.2)	0.2 (0.0, 0.3)	0.0 (−0.1, 0.1)	1.4 (1.0, 1.8)	0.1 (0.0, 0.2)	0.2 (0.1, 0.3)	0.5 (0.3, 0.8)	0.2 (0.1, 0.3)	0.2 (0.1, 0.3)	0.1 (0.0, 0.1)	0.3 (0.2, 0.5)
Shaanxi	0.0 (−0.2, 0.2)	0.4 (0.1, 0.7)	0.0 (−0.5, 0.6)	7.8 (6.9, 8.6)	0.9 (0.6, 1.1)	0.5 (0.2, 0.8)	0.5 (0.2, 0.8)	0.7 (0.4, 0.9)	1.0 (0.6, 1.4)	0.4 (0.2, 0.5)	1.2 (0.9, 1.6)
Henan	0.0 (0.0, 0.1)	0.2 (0.0, 0.4)	0.0 (−0.2, 0.2)	8.9 (8.1, 10)	0.8 (0.6, 1.1)	0.8 (0.5, 1.0)	0.3 (0.0, 0.6)	0.7 (0.5, 1.0)	0.7 (0.5, 1.1)	0.2 (0.1, 0.4)	1.2 (1.0, 1.6)
Jiangsu	0.5 (0.0, 1.1)	1.5 (1.0, 2.2)	2.7 (1.7, 3.7)	8.7 (6.7, 10.7)	1.0 (0.6, 1.6)	1.2 (0.6, 2.1)	1.4 (0.9, 2.1)	1.3 (0.9, 1.9)	1.4 (0.9, 1.9)	0.6 (0.3, 0.9)	2.0 (1.3, 2.8)
Anhui	0.7 (0.3, 1.2)	1.0 (0.6, 1.3)	0.3 (0.2, 0.5)	9.7 (8.8, 10.7)	0.6 (0.5, 0.7)	0.7 (0.4, 0.9)	0.8 (0.6, 1.1)	0.8 (0.6, 1.0)	1.3 (1.0, 1.6)	0.7 (0.5, 1.0)	1.7 (1.3, 2.0)
Shanghai	1.6 (1.1, 2.3)	9.3 (7.6, 11.2)	3.8 (2.3, 5.6)	15.6 (11.0, 20.8)	7.0 (5.9, 8.2)	6.2 (5.0, 7.7)	12.3 (10.2, 14.5)	6.9 (5.9, 8.0)	10.2 (8.7, 11.8)	9.3 (7.8, 10.7)	8.2 (6.5, 10.1)
Hubei	1.7 (0.8, 2.8)	1.3 (0.5, 2.3)	1.1 (0.7, 1.7)	6.4 (4.8, 8.0)	0.9 (0.3, 1.8)	0.3 (−0.2, 0.9)	1.5 (0.8, 2.5)	0.8 (0.3, 1.4)	1.1 (0.7, 1.7)	0.8 (0.4, 1.3)	1.6 (0.9, 2.4)
Sichuan	0.1 (−0.1, 0.2)	0.3 (0.0, 0.5)	0.2 (0.0, 0.5)	8.0 (6.8, 9.3)	0.4 (0.2, 0.7)	0.5 (0.2, 0.8)	0.3 (0.1, 0.6)	0.6 (0.2, 1.1)	0.3 (0.1, 0.6)	0.2 (0.0, 0.5)	1.1 (0.8, 1.5)
Zhejiang	2.5 (1.4, 3.7)	2.6 (2.0, 3.3)	1.3 (0.8, 1.9)	13.2 (10.5, 15.9)	3.4 (2.2, 4.8)	3.6 (2.3, 4.9)	5.8 (4.2, 7.7)	3.8 (3.0, 4.6)	6.1 (4.8, 7.6)	5.5 (4.1, 7.2)	4.8 (3.5, 6.1)
Chongqing	0.8 (0.1, 1.5)	1.1 (0.6, 1.5)	0.9 (0.3, 1.4)	5.0 (4.1, 6.0)	0.8 (0.5, 1.1)	0.5 (0.2, 0.9)	1.0 (0.6, 1.5)	0.7 (0.1, 1.3)	0.7 (0.4, 0.9)	0.6 (0.3, 0.9)	1.2 (0.7, 1.7)
Jiangxi	1.1 (0.7, 1.4)	0.4 (0.2, 0.7)	1.1 (0.7, 1.3)	7.6 (6.6, 8.6)	0.6 (0.4, 0.8)	0.3 (0.1, 0.6)	0.6 (0.2, 0.9)	0.8 (0.5, 1.1)	1.2 (0.8, 1.7)	0.6 (0.2, 0.9)	1.4 (1.0, 1.8)
Hunan	0.2 (−0.2, 0.6)	1.0 (0.5, 1.6)	0.4 (0.1, 0.8)	8.1 (7.1, 9.2)	1.1 (0.6, 1.5)	0.6 (0.3, 1.0)	0.9 (0.4, 1.4)	0.8 (0.4, 1.3)	0.8 (0.5, 1.2)	0.5 (0.2, 0.7)	1.4 (1.0, 1.9)
Guizhou	0.2 (0.1, 0.3)	0.1 (0.1, 0.2)	0.0 (0.0, 0.0)	5.4 (4.8, 6.0)	0.7 (0.6, 0.9)	0.3 (0.2, 0.4)	0.5 (0.3, 0.8)	1.0 (0.6, 1.4)	0.7 (0.5, 1.0)	0.9 (0.5, 1.3)	1.0 (0.8, 1.2)
Fujian	0.3 (−0.2, 0.7)	0.4 (0.2, 0.5)	0.2 (0.0, 0.5)	5.1 (4.1, 6.1)	1.4 (0.9, 2.0)	1.0 (0.5, 1.6)	1.5 (0.9, 2.2)	1.3 (0.9, 1.7)	1.2 (0.9, 1.7)	0.7 (0.4, 1.1)	1.3 (0.9, 1.8)
Yunnan	0.2 (−0.1, 0.5)	0.3 (0.1, 0.6)	0.3 (0.0, 0.6)	2.9 (2.1, 3.7)	0.5 (0.3, 0.9)	0.1 (−0.1, 0.2)	0.2 (0.0, 0.4)	0.1 (−0.1, 0.4)	0.2 (0.0, 0.5)	0.3 (0.1, 0.5)	0.5 (0.2, 0.8)
Guangxi	1.8 (1.3, 2.4)	0.9 (0.6, 1.2)	0.7 (0.5, 0.8)	3.9 (2.5, 5.3)	1.6 (1.0, 2.2)	0.4 (0.1, 0.7)	1.5 (1.1, 2.0)	1.0 (0.3, 1.7)	1.8 (1.2, 2.4)	1.2 (0.8, 1.6)	1.5 (0.9, 2.0)
Guangdong	2.4 (1.9, 3.0)	2.8 (2.2, 3.5)	3.6 (2.9, 4.3)	8.1 (6.0, 10.1)	4.5 (3.4, 5.4)	2.3 (1.7, 2.9)	4.8 (3.6, 5.9)	3.6 (2.6, 4.6)	4.9 (3.8, 5.9)	3.4 (2.5, 4.1)	4.0 (3.1, 5.0)
Hainan	0.5 (0.2, 0.9)	0.6 (0.1, 1.2)	0.4 (0.2, 0.6)	0.9 (0.0, 1.8)	0.6 (0.3, 1.0)	0.4 (0.2, 0.6)	0.2 (0.0, 0.4)	1.1 (0.6, 1.7)	0.4 (0.2, 0.6)	0.4 (0.1, 0.8)	0.6 (0.2, 0.9)
Nation	0.7 (0.2, 1.3)	1.1 (0.5, 1.8)	0.9 (0.3, 1.6)	7.8 (6.1, 9.7)	1.4 (0.7, 2.1)	1.0 (0.4, 1.6)	1.7 (0.8, 2.5)	1.4 (0.8, 2.1)	1.9 (1.1, 2.9)	1.2 (0.7, 1.8)	2.5 (1.5, 3.6)

The highest type/subtype‐specific influenza‐attributable ILI burden was observed in 2009, and on average 6.8 (95% CI: 5.5, 8.1) consultations per 1000 py presented because of influenza A(H1N1)pdm (Table 2). After the pandemic, seasonal influenza A(H3N2) viruses were associated with the highest ILI consultation rates (0.7 per 1000 py [95% CI: 0.4, 0.9]), followed by influenza A(H1N1)pdm and influenza B (Table 2). Influenza A(H3N2) contributed most in 2014, the year with the highest burden after the pandemic. At the national level, influenza B was associated with no more than 0.5 consultations per 1000 py over 10 years.

**Table 2:** Influenza‐associated influenza‐like illness consultations by influenza type/subtype and age group in China, 2006‐2015 (per 1000 py)

Year	A(H1N1)	A(H3N2)	A(H1N1)pdm	B	0‐14 y	15‐59 y	≥60 y	Ratio (0‐14 y: ≥60 y)	Ratio (15‐59 y: ≥60 y)
2006	0.5 (0.2, 0.9)	0.1 (0.1, 0.3)	0	0.1 (0.0, 0.3)	1.2 (−0.5, 2.9)	0.6 (0.2, 1.1)	0.5 (0.1, 0.9)	2.4	1.2
2007	0.1 (0.0, 0.1)	0.8 (0.5, 1.1)	0	0.3 (0.0, 0.6)	2.5 (0.6, 4.4)	0.9 (0.3, 1.5)	0.9 (0.3, 1.4)	2.8	1.0
2008	0.4 (0.1, 0.7)	0.3 (0.2, 0.5)	0	0.2 (0.0, 0.4)	1.8 (−0.1, 3.7)	0.8 (0.2, 1.4)	0.6 (0.1, 1.2)	3.0	1.3
2009	0.2 (0.1, 0.4)	0.7 (0.5, 1.0)	6.8 (5.5, 8.1)	0.1 (0.0, 0.2)	12.4 (6.5, 18.6)	7.9 (6.2, 9.5)	1.5 (0.1, 3.2)	8.3	5.3
2010	0	0.6 (0.3, 0.8)	0.5 (0.3, 0.6)	0.4 (0.0, 0.8)	3.2 (0.9, 5.7)	1.2 (0.5, 1.8)	0.8 (0.2, 1.4)	4.0	1.5
2011	0	0.1 (0.1, 0.1)	0.7 (0.4, 1.0)	0.2 (0.0, 0.4)	2.0 (0.3, 3.9)	0.8 (0.3, 1.4)	0.6 (0.2, 1.1)	3.3	1.3
2012	0	1.1 (0.8, 1.5)	0.1 (0.0, 0.1)	0.5 (0.0, 0.9)	3.6 (1.2, 6.3)	1.3 (0.6, 2.1)	1.1 (0.4, 1.8)	3.3	1.2
2013	0	0.4 (0.3, 0.6)	0.9 (0.5, 1.4)	0.1 (0.0, 0.2)	2.8 (0.7, 5.0)	1.2 (0.6, 1.9)	0.9 (0.4, 1.5)	3.1	1.3
2014	0	1.0 (0.6, 1.4)	0.7 (0.4, 1.0)	0.2 (0.0, 0.5)	3.8 (1.1, 6.7)	1.6 (0.8, 2.5)	1.3 (0.6, 2.0)	2.9	1.2
2015	0	0.9 (0.6, 1.2)	0.1 (0.1, 0.1)	0.3 (0.1, 0.6)	2.6 (0.8, 4.5)	1.0 (0.5, 1.6)	0.8 (0.3, 1.3)	3.2	1.2
Mean	0.3 (0.1, 0.5)	0.6 (0.4, 0.8)	1.4 (1.0, 1.7)	0.2 (0.0, 0.5)	4.5 (1.3, 7.9)	2.3 (1.4, 3.3)	1.1 (0.3, 2.0)	4.1	2.1
Mean 2010‐2015	—	0.7 (0.4, 0.9)	0.5 (0.3, 0.7)	0.3 (0.0, 0.5)	3.0 (0.8, 5.3)	1.2 (0.6, 1.9)	0.9 (0.4, 1.5)	3.3	1.3

**Figure 2:**
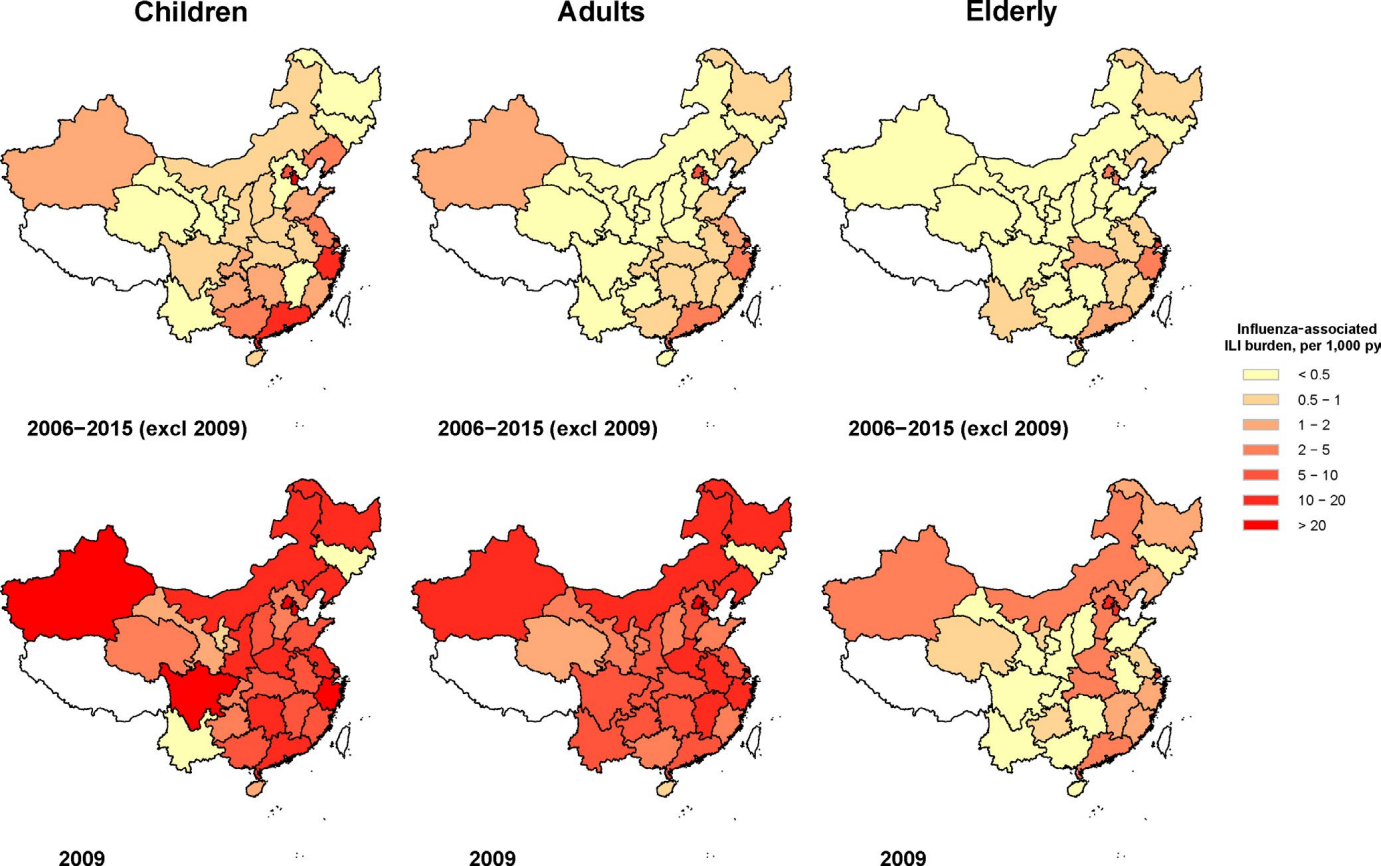
Average influenza‐associated ILI burden among children, adults and elderly in 30 provinces in China, 2006‐2015

The average influenza‐associated ILI burden was 4.5 (95% CI: 1.3, 7.9) consultations per 1000 py among children aged below 15 years old, higher than that among people aged 15‐59 years (2.3 [95% CI: 1.4, 3.3]) and elderly aged 60 years or above (1.1 [95% CI: 0.3, 2.0]). The pattern was consistent across the 10‐year period. In the pandemic year 2009, there washigher influenza burden among all ages compared with other years, and the increase was much more obvious among children and adults (Table 2, Figure 2). We also estimated the burden ratio of patients aged 0‐14 years vs ≥60 years (ratio_1_) and patients aged 15‐59 years vs ≥60 years (ratio_2_) from 2005 to 2016. We observed higher post‐pandemic ratio_1_, suggesting a shift of influenza‐attributed outpatient burden to children since 2009 (Table 2).

We estimated the proportion of type/subtype‐specific influenza‐associated ILI burden by each province and for each year (Figure S6). Though the pattern was inconsistent between provinces before 2009, the dominant burden was generally attributed to the same influenza type/subtype across 30 provinces after 2009. Most provinces had larger proportion of burden associated with influenza A(H1N1)pdm or A(H3N2) comparing to influenza B (Figure S6).

### 3.4 Cluster analysis on between‐province variation

We further explored if socioeconomic or health‐related factors could explain the variation in influenza‐associated ILI burden at province level. We plotted perGRP, perDI, perUHCMS, perRHCMS, and population density against the estimated influenza‐associated ILI consultations at each province (Figure S7). We identified significant correlations (ρ = 0.52‐0.88, all *P* < .05) for all of these variables, indicating moderate to strong positive associations. Further, a Euclidean distance matrix was constructed based on the above factors from 30 provinces and was compared with another distance matrix of the estimated influenza‐associated ILI burden from 30 provinces (Figure S8). The Mantel correlation statistic was 0.70 (*P* < .001), implying that perGRP, perDI, perUHCMS, perRHCMS, and population density could explain more than half of the heterogeneity of influenza‐associated burden across provinces.

## 4 DISCUSSION

Based on national sentinel hospital‐based ILI and virological surveillance system, we estimated a national average of 2.5 (95% CI: 1.5, 3.6) influenza‐associated consultations per 1000 py from 2006 to 2015. With a total of 3.4 (95% CI: 2.0, 4.9) million persons medically consulted on an annual basis because of influenza viruses, the overall influenza‐associated outpatient burden was substantial in China.

The pandemic year 2009 was associated with a sharp increase of influenza‐associated ILI burden, with a national mean of 7.8 (95% CI: 6.1, 9.7) consultations per 1000 py. Such increase was observed in each age group and in most provinces (Tables 1 and 2). However, the sharp increase was not observed in the United States, where total influenza‐associated ILI burden was 10.6 consultations per 1000 py in the pandemic year, compared with 1.9‐10.7 in the following three seasons.^6^ Influenza vaccination coverages in the United States and in China were about 40%‐50% and 1%‐2%, respectively.^23^, ^24^ Such difference may indicate lower baseline awareness against seasonal influenza in China, resulting in a larger change in health‐seeking behavior when the pandemic drew intense attention.^25^

Influenza‐associated ILI burden in China was constantly higher among children (0‐14 years), followed by adults (15‐59 years) and then the elderly (≥60 years). The burden was observed to shift noticeably from the elderly to adults in particular and also children in 2009. Similar pattern was reported by studies from the United States and UK,^6^, ^26^ though the shift was more pronounced in China. One possible explanation was the pre‐existing cross‐immunity against influenza A(H1N1)pdm among the older population.^27^ After the pandemic, influenza A(H3N2) remained to be the leading influenza subtype associated with most influenza‐associated ILI burden, followed by influenza A(H1N1)pdm and then influenza B. Also, the difference in influenza‐associated outpatient burden by age group could be attributed to the differences in attack rate and healthcare seeking behavior. In any case, the larger influenza‐associated outpatient burden among children and adults should prompt for shift from hospital‐based medical consultation to primary care health facilities among younger patients with ILI. This will allow better allocation of limited healthcare resources.

Comparing to the United States, China had a lower overall burden estimates but with a larger variation between provinces.^6^ The annual overall influenza burden was more than 8 consultations per 1000 py in municipalities including Beijing, Shanghai, and Tianjin and <1 consultation per 1000 py in provinces such as Ningxia, Qinghai, and Gansu. This may be explained by the variation in economic development and thus health‐seeking behavior patterns between these provinces. Especially in less developed provinces, individuals may adopt self‐medication and not present to outpatients unless symptoms were severe. Our cluster analysis confirmed this at an ecological level that heterogeneity in burden could be explained by socioeconomic factors such as GDP (per capita) and capability in health expenses (per capita). Such information could be utilized to formulate more refined or localized health policies and economic assessment.

Estimating population influenza attributable to ILI burden is challenged by defining the population covered by the surveillance system. Unlike some European countries and the United States where the registries by general practitioners were obtainable,^26^, ^28^ the population covered by each surveillance hospital was less well‐defined in China. We assumed that, first, there were limited cross‐province outpatient consultations; second, the surveillance network outpatient‐to‐population ratio is representative to the whole province. We calculated the population covered by surveillance network based on these assumptions. This methodology was proposed as an alternative to determine population covered by surveillance network for population disease burden estimation, but further evaluation is still needed. In our analysis, the method provided reasonable estimates as compared to other places and hence should have potential use in other countries without general practitioner registration system.

The influenza surveillance system in China incorporates ILI surveillance and virological surveillance from 30 provinces, enabling the estimation of influenza‐associated outpatient burden on national and provincial level. To our knowledge, this is the first estimation on influenza‐attributable outpatient burden in China at the national level. Throughout a 10‐year period, we were able to understand the disease burden dynamic and provide evidence for planning of vaccination programs and antivirals to reduce the public health impact of seasonal influenza.

In China, influenza vaccine was categorized as class II vaccine, requiring out‐of‐pocket payment. Vaccination coverage is very low in China, even in developed provinces.^24^, ^29^ The estimated vaccination coverage around our study period was about 1.5% for people aged 6 months or above.^24^ The cost per dose trivalent influenza vaccine in China is around 10 USD, roughly the daily wage in many regions in China.^30^ Though more regional governments increased influenza vaccination promotion and subsidization, only a few provinces/cities offer free vaccination for the elderly or other high‐risk population such as children, pregnant women, and persons with chronic medical problems. Other barriers to influenza vaccination in China include lower accessibility and higher distribution cost in rural area.

Our findings are subject to several limitations. Since the influenza pandemic in 2009, most network laboratories started utilizing RT‐PCR to detect influenza viruses. Afterward, some but not all provinces switched back to virus culture. Thus, potential misclassification bias might happen with variation in laboratory methods sensitivity. We tried to reduce this bias by using an adjustment ratio as in a previous study.^5^ In addition, some patients with ILI may visit general practitioner clinics or community healthcare centers and hence were not captured by our surveillance network. Primary care is still under‐utilized in China,^31^ and hence, the impact on our estimates is limited, but this trend will probably change in the future as promoting primary care is one of the major goals of the healthcare reform. The national sentinel hospital‐based ILI surveillance system in China was established considering the recommendations from the World Health Organization.^15^ This included selection of general outpatient clinics or acute care facilities as sentinel sites and inclusion of hospitals from both rural and urban areas, which should achieve better representation of the consultations. However, we could not rule out the possibility that ILI patients may be more or less likely to visit the sentinel hospitals, compared to other hospitals within the catchment area. Comparisons between provinces may also be affected by potential variation in the representativeness of sentinel hospitals. Virological surveillance data from the outpatient setting in rural or less urbanized provinces may be more subjective to variable testing practices and hence less representative.^32^ Moreover, we do not have data on other respiratory virus, for example, respiratory syncytial virus, which may co‐circulated with influenza viruses at the same time. Finally, the surveillance network has been expanded in response to the 2009 pandemic. We observed increase of burden of post‐pandemic period compared with pre‐pandemic. However, we were not able to determine whether this elevation was associated the expansion of surveillance network.

## 5 PUBLIC HEALTH IMPLICATIONS

Influenza vaccination is the most effective tool in preventing seasonal influenza. This study highlights provinces with higher influenza outpatient burden where influenza vaccination is likely to be more cost‐effective. Our findings also suggest the importance of increasing vaccination coverage among children, of whom influenza‐associated outpatient burden was the highest but was not included as priority group for most regions. Continued monitoring of influenza‐associated disease burden would be important for further planning and evaluating the cost‐effectiveness of influenza vaccination program.^33^, ^34^, ^35^

Furthermore, influenza surveillance should also extend to primary care settings including general practitioner clinics or community healthcare centers in the future. This could give a full picture of influenza‐associated medical seeking behaviors, outpatient burden, and impact of influenza epidemics in the community, also necessary for pandemic influenza preparedness.

In conclusion, we found substantial influenza‐associated outpatient burden in 30 provinces in China across 10‐year period. The burden varied by time, increased sharply in 2009 pandemic, and was more pronounced in well‐developed provinces and among children, and influenza A(H3N2) contributed to most outpatient burden after the 2009 pandemic. Our study informs the planning of influenza vaccination program in China.

## CONFLICT OF INTEREST

BJC has received research funding from Sanofi Pasteur for a study of influenza vaccine effectiveness. The authors report no other potential conflicts of interest.

## ETHICS APPROVAL AND CONSENT TO PARTICIPATE

This project was therefore determined by China CDC not to be subject to institutional review board approval.

## Data Availability

The datasets used and/or analyzed during the current study are available from the corresponding author on reasonable request.
