# Supplementary material for: Burden of influenza‐associated outpatient influenza‐like illness consultations in China, 2006‐2015: A population‐based study
Source: Influenza Other Respir Viruses. 2019 Dec 23;14(2):162–72. doi: 10.1111/irv.12711 (PMC7040965; doi:10.1111/irv.12711)
Supplement: Supplementary file 1 [file IRV-14-162-s001.docx]

**Supplementary Appendix**

**Data cleaning and weighting**

The objective of data cleaning and weighting is to improve data validity and representativeness. For ILI surveillance data, firstly, we removed surveillance records of off-surveillance period (from April to August every year before 2009) from 12 provinces conducting winter surveillance. Secondly, we evaluated data validity by each surveillance hospital. The factors taken into consideration included the completeness of reporting, consistency between reporting department and patient ages, and consistency between number of ILI consultations and number of total consultations. All hospitals were evaluated based on the same scoring criteria according to the above factors. Scores of “absence of reporting” and “incomplete reporting” for each hospital were added up on annual basis, and the corresponding records from those hospitals which scored above a pre-defined threshold in a year were removed from further analysis (5.6% of the observations removed). Moreover, we considered the consistency between the reporting department and patient ages. We removed the corresponding records from paediatric clinics if adult ILI patients were included in their reporting. The corresponding records from a hospital were also removed when such inconsistencies were identified frequently in a year above a tolerance threshold (0.8% of the observations removed). Thirdly, to improve representativeness of the surveillance population to underlying population structure, we calculated the overall ILI rate by weighting the ILI consultation rate from paediatrics and medical departments using the proportion of population age below and above 15 years for each province in weekly basis:

$${ILI rate}_{\mathrm{overall}}={ILI rate}_{\mathrm{pediatrics}}\times\mathrm{prop}_{pop<15y}+{ILI rate}_{\mathrm{medicine}}\times\mathrm{prop}_{pop\geq15y}$$

For virological data, we also removed recordings from off-surveillance period, and extreme values of weekly influenza virus positive rate. As mentioned above, RT-PCR has been utilized since May 2009 in response to 2009 influenza pandemic in some provinces. To account for the different sensitivity to influenza detection between RT-PCR and virus culture, we adjusted specimens that were tested positive by RT-PCR using an adjustment ratio based on a previous study (1). Finally, specimen identified as influenza A which was unsubtyped were imputed by the ratio of characterized influenza A(H1N1), A(H3N2) and A(H1N1)pdm in the same province and same week.

**Comparison before and after data cleaning**

Figure S1 and S2 show weekly province-specific ILI consultation rates (proportion of ILI consultation among total consultations) and laboratory confirmed virus positive rates (proportion of virus test-positive for influenza among all specimens) before and after data cleaning and weighting (Figure S1, S2). Noticeable improvement in data stability can be observed in some provinces.

**Generalized additive model**

We estimated influenza-associated ILI burden using generalized additive regression models by the following equation:

$$E\left( \frac{{Total ILI}_{t} \mathrm{consultations}}{\mathrm{Population}_{t}} \right)=\beta_{A\left( H1N1 \right)}\mathrm{Flu}_{A\left( H1N1 \right), t}+\beta_{A\left( H3N2 \right)}\mathrm{Flu}_{A\left( H3N2 \right), t}+\beta_{A\left( H1N1pdm \right)}\mathrm{Flu}_{A\left( H1N1pdm \right), t}+\beta_{B}\mathrm{Flu}_{B, t}+\beta_{\mathrm{Pandemic}}\mathrm{Pandemic}_{t}+\beta_{\mathrm{Pandemic}\times A\left( H1N1pdm \right)}\mathrm{Pandemic}_{t}\times\mathrm{Flu}_{H1N1pdm, t}+s\left( \mathrm{Temp}_{t},df \right)+s\left( \mathrm{AH}_{t},df \right)+s\left( t,df \right)$$

where $\mathrm{Flu}_{A\left( H1N1 \right), t}$, $\mathrm{Flu}_{A\left( H3N2 \right),t}$, $\mathrm{Flu}_{A\left( H1N1pdm \right),t}$ and $\mathrm{Flu}_{B,t}$ are denoted as virus detection rates of influenza A(H1N1), A(H3N2), A(H1N1pdm) and B in week *t*, respectively. $\mathrm{Pandemic}_{t}$refers to the dummy variable indicating the pandemic period, and $\mathrm{Pandemic}_{t}\times\mathrm{Flu}_{H1N1pdm,t}$ refers to the interaction term between pandemic period and the A(H1N1pdm) detection rate, adjusting for changes in health seeking behaviour during pandemic period because of influenza A(H1N1pdm). $s\left( \mathrm{Temp}_{t},\mathrm{df}_{\text{Temp}} \right)$, $s\left( \mathrm{AH}_{t},\mathrm{df}_{\text{AH}} \right)$ and $s\left( t,\mathrm{df}_{\text{t}} \right)$ represent the cubic splines terms on mean weekly temperature, absolute humidity and the calendar time, with the degree of freedom (df). The df for temperature, absolute humidity and calendar week were all set as 20, allowing for 2 knots each year to sufficiently describe influenza seasonal patterns. Absolute humidity was calculated according to the previous study based on relative humidity and temperature, using the Bolton’s conversion formula (2). The 95% confidence intervals (CI) for influenza-associated ILI burden were estimated using a bootstrap approach, assuming AR(2) process in each province by evaluating the autocorrelation of the residues. A separated model was fitted for each province, age group (0-14, 15-59, ≥60 years) and for overall. The 95% confidence intervals (CI) of the estimates were obtained using a bootstrap approach.

**Sensitivity analysis**

We have conducted sensitivity analysis to examine whether the expansion of network would affect the influenza-associated outpatient burden estimates by each province. In the sensitivity analysis, dummy variables were introduced allowing for changes after the influenza pendemic period till the end of study period. The model is described by the following equation:

$$E\left( \frac{{Total ILI}_{t} \mathrm{consultations}}{\mathrm{Population}_{t}} \right)=\beta_{A\left( H1N1 \right)}\mathrm{Flu}_{A\left( H1N1 \right), t}+\beta_{A\left( H3N2 \right)}\mathrm{Flu}_{A\left( H3N2 \right), t}+\beta_{A\left( H1N1pdm \right)}\mathrm{Flu}_{A\left( H1N1pdm \right), t}+\beta_{B}\mathrm{Flu}_{B, t}+\beta_{\mathrm{Pandemic}}\mathrm{Pandemic}_{t}+\beta_{\mathrm{Post}\text{-}\mathrm{pandemic}}{\mathrm{Post}\text{-}\mathrm{pandemic}}_{t}+\beta_{\mathrm{Pandemic}\times A\left( H1N1pdm \right)}\mathrm{Pandemic}_{t}\times\mathrm{Flu}_{H1N1pdm, t}+\beta_{\mathrm{Post}\text{-}\mathrm{pandemic}\times A\left( H1N1pdm \right)}{\mathrm{Post}\text{-}\mathrm{pandemic}}_{t}\times\mathrm{Flu}_{H1N1pdm, t}+\beta_{\mathrm{Post}\text{-}\mathrm{pandemic}\times A\left( H3N2 \right)}{\mathrm{Post}\text{-}\mathrm{pandemic}}_{t}\times\mathrm{Flu}_{H3N2, t} +\beta_{\mathrm{Post}\text{-}\mathrm{pandemic}\times B}{\mathrm{Post}\text{-}\mathrm{pandemic}}_{t}\times\mathrm{Flu}_{B, t} +s\left( \mathrm{Temp}_{t},df \right)+s\left( \mathrm{AH}_{t},df \right)+s\left( t,df \right)$$

where ${\mathrm{Post}\text{-}\mathrm{pandemic}}_{t}$refers to the dummy variable indicating the post-pandemic period when surveillance system has been expanded. ${\mathrm{Post}\text{-}\mathrm{pandemic}}_{t}\times\mathrm{Flu}_{H3N2,t}$ , ${\mathrm{Post}\text{-}\mathrm{pandemic}}_{t}\times\mathrm{Flu}_{H1N1pdm,t}$ , and ${\mathrm{Post}\text{-}\mathrm{pandemic}}_{t}\times\mathrm{Flu}_{B,t}$ refer to the interaction term between post-pandemic period and the virus detection rate, adjusting for changes in surveillance scale and laboratory methods. Table S1 shows the results by sensitivity analysis, similar with main analysis. The national-wide overall estimated influenza-associated ILI consultation was 2.8 per 1,000 population (95% CI: 1.6, 4.0), which was similar to that from the main analysis. Moreover, we did not observe a shift in ranking of outpatient burden by 30 provinces using Wilcoxon Signed-Rank Test (p = 0.609).

**Cluster analysis on between-province variation**

To understand the factors that could explain the between-province variation of influenza-associated ILI burden, we examined the correlation between the estimated influenza-associated ILI burden and socioeconomic factors across 30 provinces. Factors significantly (p<0.05) correlated with influenza-associated ILI burden were included in a cluster analysis, and a Euclidean distance matrix summarizing those factors was built. Another distance matrix on influenza-associated ILI burden estimates was built independently based on the same method. We hypothesized that if the factors could explain between-province variation, the two clusters would be comparable. Mantel R statistic was used to examine the comparability between clusters (3).

**Reference:**

1. Yu H, Feng L, Viboud CG, Shay DK, Jiang Y, Zhou H, et al. Regional variation in mortality impact of the 2009 A (H1N1) influenza pandemic in China. Influenza and Other Respiratory Viruses 2013;7(6):1350-1360.

2. Bolton D. The computation of equivalent potential temperature. Monthly weather review 1980;108(7):1046-1053.

3. Mantel N. The detection of disease clustering and a generalized regression approach. Cancer Research 1967;27(2):209-20.

Table S1. Demographic characteristics, influenza surveillance and laboratory test results in 30 provinces in China, 2006-2015.

| Province | Population  in 2013  (1,000,000) | per capita GRP in 2015($) | *n* of hospital in 2015 | *n* of hospital beds in 2015 (per 1,000) | *n* of outpatient department in 2015 | per capita Income in 2015($) | per capita health expense in 2012($) | | Mean annual *n* of total consultations  (1,000) | Mean annual ILI consultation rate | | Mean annual specimen tested | | Mean annual sample positive for influenza | | Mean annual influenza positive rate (per 1,000) | |
| --- | --- | --- | --- | --- | --- | --- | --- | --- | --- | --- | --- | --- | --- | --- | --- | --- | --- |
|  |  |  |  |  |  |  | **Urban** | **Rural** |  | **2006-15 (excl 2009)** | **2009** | **2006-15 (excl 2009)** | **2009** | **2006-15 (excl 2009)** | **2009** | **2006-15 (excl 2009)** | **2009** |
| Heilongjiang | 38.1 | 5,719 | 671 | 558 | 5,242 | 2,695 | 171 | 105 | 1,609 | 2.3% | 9.3% | 7,210 | 6,707 | 546 | 929 | 9.0% | 13.8% |
| Xinjiang | 23.6 | 5,803 | 705 | 637 | 4,909 | 2,443 | 149 | 64 | 1,943 | 3.3% | 11.2% | 7,078 | 6,773 | 435 | 1,088 | 6.3% | 16.1% |
| Inner Mongolia | 25.1 | 10,305 | 409 | 533 | 6,261 | 3,233 | 196 | 85 | 1,129 | 3.4% | 7.6% | 4,684 | 6,629 | 209 | 450 | 5.6% | 6.8% |
| Liaoning | 43.8 | 9,472 | 610 | 609 | 11,164 | 3,562 | 190 | 80 | 3,576 | 4.2% | 6.1% | 8,682 | 10,935 | 628 | 1,126 | 8.7% | 10.3% |
| Jilin | 27.5 | 7,404 | 353 | 525 | 7,979 | 2,708 | 210 | 122 | 1,213 | 1.9% | 1.0% | 4,625 | 4,201 | 373 | 561 | 10.3% | 13.4% |
| Beijing | 21.7 | 15,435 | 295 | 514 | 4,238 | 7,023 | 240 | 163 | 5,188 | 3.9% | 5.2% | 7,211 | 7,029 | 1,092 | 1,623 | 18.40% | 23.1% |
| Tianjin | 15.5 | 15,647 | 272 | 412 | 1,462 | 4,535 | 226 | 110 | 2,847 | 8.1% | 13.5% | 3,408 | 3,283 | 576 | 890 | 17.7% | 27.1% |
| Hebei | 74.2 | 5,834 | 1041 | 461 | 11,922 | 2,626 | 152 | 79 | 1,909 | 2.2% | 4.9% | 7,312 | 11,243 | 626 | 1,811 | 8.7% | 16.1% |
| Ningxia | 6.7 | 6,349 | 112 | 506 | 1,178 | 2,512 | 154 | 71 | 804 | 0.5% | 3.6% | 3,140 | 4,002 | 247 | 422 | 8.7% | 10.6% |
| Shanxi | 36.6 | 5,061 | 654 | 500 | 8,591 | 2,588 | 131 | 71 | 1,372 | 2.4% | 3.5% | 4,658 | 4,297 | 590 | 854 | 14.4% | 19.9% |
| Gansu | 26.0 | 3,792 | 278 | 491 | 6,732 | 1,952 | 152 | 58 | 1,015 | 1.8% | 3.3% | 6,566 | 10,009 | 613 | 1,503 | 10.0% | 15.0% |
| Shandong | 98.5 | 9,300 | 1217 | 527 | 15,321 | 3,290 | 146 | 92 | 2,471 | 1.8% | 3.5% | 9,696 | 8,002 | 998 | 2,255 | 12.2% | 28.2% |
| Qinghai | 5.9 | 5,979 | 112 | 587 | 754 | 2,292 | 131 | 75 | 335 | 1.3% | 2.8% | 2,442 | 1,084 | 129 | 111 | 8.1% | 10.3% |
| Shaanxi | 37.9 | 6,903 | 704 | 559 | 6,177 | 2,521 | 176 | 90 | 998 | 3.2% | 6.4% | 5,514 | 4,649 | 440 | 973 | 12.1% | 20.9% |
| Henan | 94.8 | 5,670 | 951 | 516 | 6,794 | 2,482 | 157 | 68 | 2,390 | 3.2% | 5.8% | 7,304 | 11,355 | 568 | 2,473 | 7.4% | 21.8% |
| Jiangsu | 79.8 | 12,753 | 1013 | 519 | 9,633 | 4,281 | 153 | 105 | 5,712 | 4.2% | 5.9% | 17,367 | 19,499 | 1,443 | 4,783 | 11.6% | 24.5% |
| Anhui | 61.4 | 5,217 | 670 | 435 | 3,422 | 2,661 | 166 | 74 | 1,889 | 4.6% | 8.0% | 11,784 | 10,185 | 1,104 | 1,811 | 9.3% | 17.8% |
| Shanghai | 24.1 | 15,044 | 181 | 508 | 2,174 | 7,227 | 147 | 149 | 6,088 | 1.7% | 2.2% | 10,370 | 8,452 | 1,918 | 1,910 | 18.2% | 22.6% |
| Hubei | 58.5 | 7,341 | 532 | 586 | 7,411 | 2,902 | 149 | 86 | 2,676 | 4.7% | 3.9% | 9,360 | 9,487 | 1,006 | 2,487 | 11.6% | 26.2% |
| Sichuan | 82.0 | 5,330 | 1291 | 596 | 14,897 | 2,496 | 112 | 72 | 2,985 | 1.8% | 5.4% | 8,288 | 9,732 | 716 | 1,891 | 9.2% | 19.4% |
| Zhejiang | 55.4 | 11,253 | 485 | 492 | 10,345 | 5,151 | 178 | 108 | 5,943 | 3.5% | 5.2% | 9,144 | 11,489 | 1,424 | 2,894 | 13.3% | 25.2% |
| Chongqing | 30.2 | 7,583 | 439 | 585 | 6,269 | 2,915 | 160 | 70 | 1,463 | 1.0% | 2.2% | 3,612 | 3,999 | 466 | 915 | 14.4% | 22.9% |
| Jiangxi | 45.7 | 5,323 | 364 | 433 | 4,168 | 2,672 | 97 | 55 | 1,787 | 3.6% | 6.7% | 7,634 | 7,061 | 1,015 | 1,434 | 12.1% | 20.3% |
| Hunan | 67.8 | 6,197 | 703 | 585 | 10,792 | 2,800 | 133 | 72 | 2,570 | 4.3% | 8.1% | 11,692 | 15,187 | 1,000 | 3,663 | 9.2% | 24.1% |
| Guizhou | 35.3 | 4,326 | 920 | 556 | 3,277 | 1,985 | 95 | 41 | 891 | 5.0% | 8.0% | 6,888 | 6,685 | 654 | 953 | 6.8% | 14.3% |
| Fujian | 38.4 | 9,851 | 348 | 451 | 5,460 | 3,682 | 112 | 55 | 5,044 | 2.6% | 3.7% | 7,618 | 10,443 | 931 | 2,684 | 11.6% | 25.7% |
| Yunnan | 47.4 | 4,175 | 725 | 501 | 6,611 | 2,206 | 136 | 53 | 1,943 | 2.7% | 5.2% | 9,295 | 7,573 | 553 | 1,076 | 6.9% | 14.2% |
| Guangxi | 48.0 | 5,100 | 325 | 447 | 9,255 | 2,446 | 128 | 56 | 2,534 | 5.4% | 7.8% | 8,632 | 7,278 | 880 | 1,403 | 9.1% | 19.3% |
| Guangdong | 108.5 | 9,783 | 793 | 402 | 14,067 | 4,038 | 152 | 65 | 7,695 | 4.0% | 5.0% | 16,937 | 19,013 | 1,812 | 3,831 | 10.0% | 20.1% |
| Hainan | 9.1 | 5,916 | 155 | 425 | 1,578 | 2,751 | 144 | 44 | 553 | 2.8% | 4.3% | 3,119 | 4,185 | 175 | 373 | 6.4% | 8.9% |
| Nation | 1367.6 | 7,672 | 17,328 | 512 | 208,083 | 3,185 | 150 | 79 | 78,572 | 3.1% | 5.5% | 231,276 | 250,466 | 23,162 | 49,176 | 10.0% | 19.6% |

Table S2. Sensitivity analysis on influenza associated influenza-like illness consultations in 30 provinces in China, 2006-2015 (per 1,000 populations).

|  | 2006 | | 2007 | | 2008 | | 2009 | | 2010 | | 2011 | | 2012 | | 2013 | | 2014 | | 2015 | | Mean | | |
| --- | --- | --- | --- | --- | --- | --- | --- | --- | --- | --- | --- | --- | --- | --- | --- | --- | --- | --- | --- | --- | --- | --- | --- |
| Heilongjiang | 0.6 | (0.0, 1.2) | 1.2 | (0.6, 1.9) | 0.9 | (0.3, 1.4) | 11.3 | (9.8, 13.0) | 0.4 | (-0.3, 1.3) | 0.3 | (-0.1, 0.8) | 0.2 | (-1.0, 1.7) | 0.6 | (-0.1, 1.6) | 1.2 | (-0.1, 2.7) | 0.3 | (-0.6, 1.5) | 1.7 | (0.8, 2.7) |  |
| Xinjiang | 0.6 | (0.1, 1.3) | 0.2 | (-0.1, 0.6) | 0.5 | (0.0, 1.0) | 25.2 | (21.9, 28.0) | 1.0 | (0.3, 1.8) | 0.7 | (0.2, 1.2) | 0.8 | (-0.1, 1.9) | 1.5 | (0.6, 2.5) | 1.7 | (0.2, 3.7) | 0.5 | (-0.1, 1.2) | 3.2 | (2.3, 4.3) |  |
| Inner Mongolia | 0.1 | (0.0, 0.1) | 0.5 | (-0.2, 1.4) | 0.4 | (0.1, 0.6) | 15.5 | (12.9, 18.3) | 0.7 | (0.0, 1.4) | 0.2 | (-0.2, 0.7) | 0.6 | (-0.3, 1.7) | 0.3 | (-0.3, 1.1) | 0.6 | (-0.3, 1.5) | 0.3 | (-0.2, 0.9) | 1.9 | (1.2, 2.8) |  |
| Liaoning | 0.8 | (0.5, 1.0) | 1.4 | (0.9, 1.8) | 0.5 | (0.3, 0.6) | 9.8 | (9.1, 10.6) | 0.8 | (0.5, 1.1) | 0.2 | (0.1, 0.3) | 0.9 | (0.4, 1.4) | 1.3 | (0.9, 1.7) | 1.6 | (1.1, 2.2) | 0.9 | (0.5, 1.3) | 1.8 | (1.4, 2.2) |  |
| Jilin | 0.1 | (-0.0, 0.3) | 0.2 | (-0.3, 0.7) | 0.3 | (-0.1, 0.6) | -1.0 | (-1.7, -0.2) | 0.1 | (-0.4, 0.7) | 0.2 | (-0.0, 0.4) | 0.1 | (-0.3, 0.6) | 0.1 | (-0.1, 0.4) | 0.2 | (-0.2, 0.6) | 0 | (-0.2, 0.3) | 0 | (-0.3, 0.5) |  |
| Beijing | 2.9 | (1.8, 4.0) | 2.9 | (1.5, 4.2) | 2.1 | (0.7, 3.3) | 29.7 | (25.2, 33.6) | 10.6 | (8.3, 12.8) | 3.7 | (3.1, 4.4) | 8.3 | (6.5, 10.2) | 9.0 | (7.5, 10.4) | 16.5 | (13.5, 19.5) | 7.7 | (5.5, 9.9) | 9.3 | (7.3, 11.3) |  |
| Tianjin | 5.8 | (4.1, 7.4) | 8.5 | (5.8, 11.2) | 9.7 | (7.2, 12.1) | 30.8 | (24.8, 35.3) | 5.6 | (3.5, 7.8) | 4.9 | (3.2, 6.2) | 7.6 | (4.0, 11.2) | 5.5 | (3.9, 7.0) | 7.1 | (4.9, 9.3) | 3.8 | (1.9, 5.7) | 8.9 | (6.3, 11.3) |  |
| Hebei | 0.1 | (-0.1, 0.4) | 0.3 | (0.0, 0.6) | 0.1 | (-0.1, 0.3) | 7.0 | (6.2, 7.8) | 0.4 | (0.2, 0.7) | 0.3 | (0.2, 0.5) | 0.6 | (0.1, 1.1) | 0.5 | (0.3, 0.9) | 0.7 | (0.4, 1.1) | 0.2 | (0.0, 0.4) | 1.0 | (0.7, 1.4) |  |
| Ningxia | 0 | (-0.1, 0.1) | 0.1 | (-0.1, 0.2) | -0.1 | (-0.6, 0.4) | 2.0 | (1.4, 2.8) | 0.1 | (-0.1, 0.4) | 0.1 | (-0.1, 0.4) | 0 | (-0.5, 0.6) | 0.2 | (-0.1, 0.4) | 0.1 | (-0.2, 0.4) | -0.1 | (-0.2, 0.1) | 0.3 | (-0.1, 0.6) |  |
| Shanxi | 0 | (-0.0, 0.1) | 0.3 | (0.0, 0.5) | 0.2 | (-0.1, 0.6) | 3.5 | (2.9, 4.1) | 0.8 | (0.4, 1.2) | 0.4 | (0.3, 0.6) | 0.8 | (0.3, 1.2) | 0.5 | (0.3, 0.7) | 0.7 | (0.4, 1.0) | 0.2 | (0.1, 0.4) | 0.8 | (0.5, 1.1) |  |
| Gansu | 0 | (-0.1, 0.2) | -0.1 | (-0.3, 0.2) | 0 | (-0.1, 0.2) | 3.3 | (2.7, 3.9) | 0.3 | (0.1, 0.5) | 0.1 | (-0.0, 0.3) | 0.3 | (-0.0, 0.6) | 0.2 | (0.0, 0.3) | 0.3 | (0.1, 0.6) | 0.1 | (-0.0, 0.3) | 0.5 | (0.2, 0.7) |  |
| Shandong | 0 | (-0.1, 0.2) | 1.0 | (0.6, 1.5) | 0.4 | (0.2, 0.7) | 2.6 | (1.9, 3.5) | 1.3 | (0.7, 1.8) | 0.9 | (0.6, 1.2) | 1.0 | (0.5, 1.4) | 1.0 | (0.7, 1.4) | 1.1 | (0.8, 1.5) | 0.8 | (0.5, 1.1) | 1.0 | (0.6, 1.4) |  |
| Qinghai | 0.1 | (-0.0, 0.2) | 0.1 | (-0.1, 0.3) | 0 | (-0.1, 0.1) | 1.4 | (1.1, 1.9) | 0.2 | (0.1, 0.4) | 0.3 | (0.1, 0.4) | 0.8 | (0.4, 1.2) | 0.2 | (0.1, 0.4) | 0.3 | (0.1, 0.5) | 0.1 | (0.0, 0.2) | 0.4 | (0.2, 0.6) |  |
| Shaanxi | 0 | (-0.2, 0.2) | 0.1 | (-0.2, 0.5) | 0.2 | (-0.4, 0.8) | 7.8 | (6.8, 8.9) | 1.1 | (0.7, 1.5) | 0.6 | (0.3, 0.9) | 0.8 | (0.4, 1.3) | 0.7 | (0.5, 1.0) | 1.3 | (0.8, 1.7) | 0.5 | (0.3, 0.7) | 1.3 | (0.9, 1.8) |  |
| Henan | 0 | (-0.1, 0.1) | 0.2 | (-0.2, 0.5) | -0.2 | (-0.4, 0.0) | 9.5 | (8.8, 10.5) | 0.7 | (0.5, 1.0) | 0.8 | (0.6, 1.1) | 0.3 | (-0.0, 0.6) | 0.7 | (0.5, 1.0) | 0.5 | (0.2, 0.9) | 0.1 | (-0.1, 0.3) | 1.3 | (1.0, 1.6) |  |
| Jiangsu | 0.5 | (-0.1, 1.1) | 1.0 | (0.4, 1.7) | 2.0 | (1.1, 3.1) | 9.1 | (6.8, 11.8) | 1.7 | (0.8, 2.8) | 1.1 | (0.1, 2.2) | 3.5 | (1.8, 5.2) | 2.0 | (1.4, 2.6) | 2.2 | (1.4, 2.9) | 1.4 | (0.6, 2.1) | 2.5 | (1.4, 3.6) |  |
| Anhui | 0.4 | (0.1, 0.7) | 0.8 | (0.4, 1.3) | 0.2 | (0.1, 0.4) | 10.9 | (9.8, 12.2) | 0.7 | (0.5, 0.9) | 0.7 | (0.5, 1.0) | 0.9 | (0.5, 1.3) | 0.8 | (0.6, 1.0) | 1.3 | (0.9, 1.7) | 0.8 | (0.4, 1.2) | 1.8 | (1.4, 2.2) |  |
| Shanghai | 1.5 | (0.9, 2.1) | 5.2 | (3.1, 7.6) | 2.6 | (1.0, 4.2) | 26.8 | (18.9, 33.3) | 9.2 | (7.8, 10.5) | 7.5 | (6.1, 9.0) | 15.4 | (12.7, 18.0) | 7.7 | (6.6, 9.1) | 11.9 | (10.1, 13.7) | 11.1 | (9.4, 12.9) | 9.9 | (7.7, 12.0) |  |
| Hubei | 1.2 | (0.3, 2.1) | 1.4 | (0.3, 2.4) | 1.1 | (0.6, 1.6) | 9.3 | (7.5, 10.9) | 1.1 | (0.3, 2.0) | 0.7 | (0.1, 1.3) | 1.2 | (0.2, 2.3) | 0.7 | (0.2, 1.2) | 0.8 | (0.3, 1.4) | 0.6 | (0.1, 1.2) | 1.8 | (1.0, 2.6) |  |
| Sichuan | 0 | (-0.1, 0.2) | 0 | (-0.2, 0.3) | 0 | (-0.2, 0.3) | 9.4 | (7.8, 10.9) | 0.9 | (0.4, 1.5) | 0.5 | (0.2, 0.9) | 0.9 | (0.3, 1.4) | 0.7 | (0.3, 1.2) | 0.8 | (0.3, 1.3) | 0.7 | (0.2, 1.1) | 1.4 | (0.9, 1.9) |  |
| Zhejiang | 2.1 | (1.0, 3.2) | 4.7 | (3.6, 5.9) | 1.3 | (0.9, 1.8) | 17.5 | (13.8, 20.7) | 3.4 | (2.3, 4.5) | 3.8 | (2.6, 5.2) | 5.0 | (3.3, 6.7) | 3.4 | (2.6, 4.3) | 5.6 | (4.2, 7.1) | 4.7 | (3.2, 6.3) | 5.2 | (3.8, 6.6) |  |
| Chongqing | 0.6 | (-0.0, 1.2) | 0.8 | (0.1, 1.4) | 0.6 | (0.1, 1.2) | 5.8 | (4.8, 6.8) | 1.0 | (0.5, 1.4) | 0.5 | (0.1, 0.9) | 1.4 | (0.6, 2.0) | 0.7 | (0.1, 1.3) | 0.9 | (0.4, 1.3) | 0.8 | (0.4, 1.2) | 1.3 | (0.7, 1.9) |  |
| Jiangxi | 1.1 | (0.7, 1.5) | 0.5 | (0.1, 0.9) | 1.1 | (0.7, 1.5) | 8.3 | (6.9, 9.5) | 0.6 | (0.3, 0.9) | 0.3 | (0.0, 0.6) | 0.6 | (0.1, 1.0) | 0.8 | (0.5, 1.1) | 1.2 | (0.7, 1.7) | 0.5 | (0.1, 1.0) | 1.5 | (1.0, 2.0) |  |
| Hunan | 0.2 | (-0.1, 0.5) | 0.8 | (0.1, 1.5) | 0.4 | (0.1, 0.7) | 10.6 | (9.3, 11.7) | 1.2 | (0.8, 1.5) | 0.6 | (0.2, 0.9) | 1.0 | (0.5, 1.4) | 0.8 | (0.4, 1.2) | 0.9 | (0.5, 1.3) | 0.5 | (0.3, 0.8) | 1.7 | (1.2, 2.1) |  |
| Guizhou | 0 | (-0.1, 0.2) | 0.1 | (0.0, 0.2) | 0 | (-0.0, 0.0) | 7.5 | (6.8, 8.2) | 0.9 | (0.7, 1.1) | 0.2 | (0.2, 0.3) | 0.6 | (0.4, 0.9) | 0.9 | (0.6, 1.2) | 0.6 | (0.4, 0.9) | 0.9 | (0.5, 1.3) | 1.2 | (0.9, 1.4) |  |
| Fujian | 0.4 | (-0.1, 0.9) | 0.4 | (0.1, 0.7) | 0.3 | (-0.1, 0.6) | 6.7 | (5.2, 8.1) | 1.5 | (0.9, 2.1) | 0.8 | (0.3, 1.5) | 1.3 | (0.6, 2.2) | 1.2 | (0.9, 1.7) | 1.1 | (0.7, 1.6) | 0.6 | (0.3, 1.1) | 1.4 | (0.9, 2.0) |  |
| Yunnan | 0.3 | (-0.0, 0.6) | 0.5 | (0.2, 0.8) | 0.4 | (0.1, 0.7) | 3.9 | (2.4, 5.1) | 0.3 | (-0.1, 0.8) | -0.1 | (-0.3, 0.2) | -0.1 | (-0.4, 0.3) | -0.1 | (-0.3, 0.2) | 0 | (-0.3, 0.3) | 0 | (-0.3, 0.4) | 0.5 | (0.1, 1.0) |  |
| Guangxi | 1.6 | (1.1, 2.1) | 1.0 | (0.7, 1.4) | 0.6 | (0.5, 0.8) | 10.9 | (8.7, 12.8) | 2.9 | (2.1, 3.7) | 0.5 | (0.1, 0.8) | 1.8 | (1.1, 2.3) | 1.1 | (0.4, 1.8) | 1.7 | (1.0, 2.3) | 1.5 | (1.0, 1.9) | 2.4 | (1.7, 3.0) |  |
| Guangdong | 2.3 | (1.8, 3.0) | 3.4 | (2.4, 4.3) | 3.7 | (3.0, 4.6) | 12.0 | (9.5, 14.4) | 4.3 | (3.1, 5.5) | 2.2 | (1.6, 2.9) | 4.3 | (2.9, 5.7) | 3.4 | (2.4, 4.4) | 4.5 | (3.3, 5.7) | 3.0 | (2.1, 4.0) | 4.3 | (3.2, 5.4) |  |
| Hainan | 0.5 | (0.2, 0.8) | 0.3 | (-0.2, 0.8) | 0.3 | (0.1, 0.5) | 3.1 | (2.0, 4.0) | 1.0 | (0.5, 1.6) | 0.4 | (0.1, 0.8) | 0.5 | (0.1, 0.9) | 1.3 | (0.7, 1.9) | 0.7 | (0.4, 1.0) | 1.1 | (0.5, 1.7) | 0.9 | (0.4, 1.4) |  |
| Nation | 0.7 | (0.1, 1.2) | 1.1 | (0.3, 2.0) | 0.9 | (0.2, 1.6) | 9.5 | (7.4, 11.7) | 1.6 | (0.7, 2.5) | 1.0 | (0.4, 1.7) | 1.8 | (0.7, 3.0) | 1.4 | (0.7, 2.2) | 2.0 | (0.9, 3.1) | 1.3 | (0.5, 2.1) | 2.8 | (1.6, 4.0) |  |

Figure S1. ILI consultation rate before and after data cleaning and weighting, 2006 to 2015.


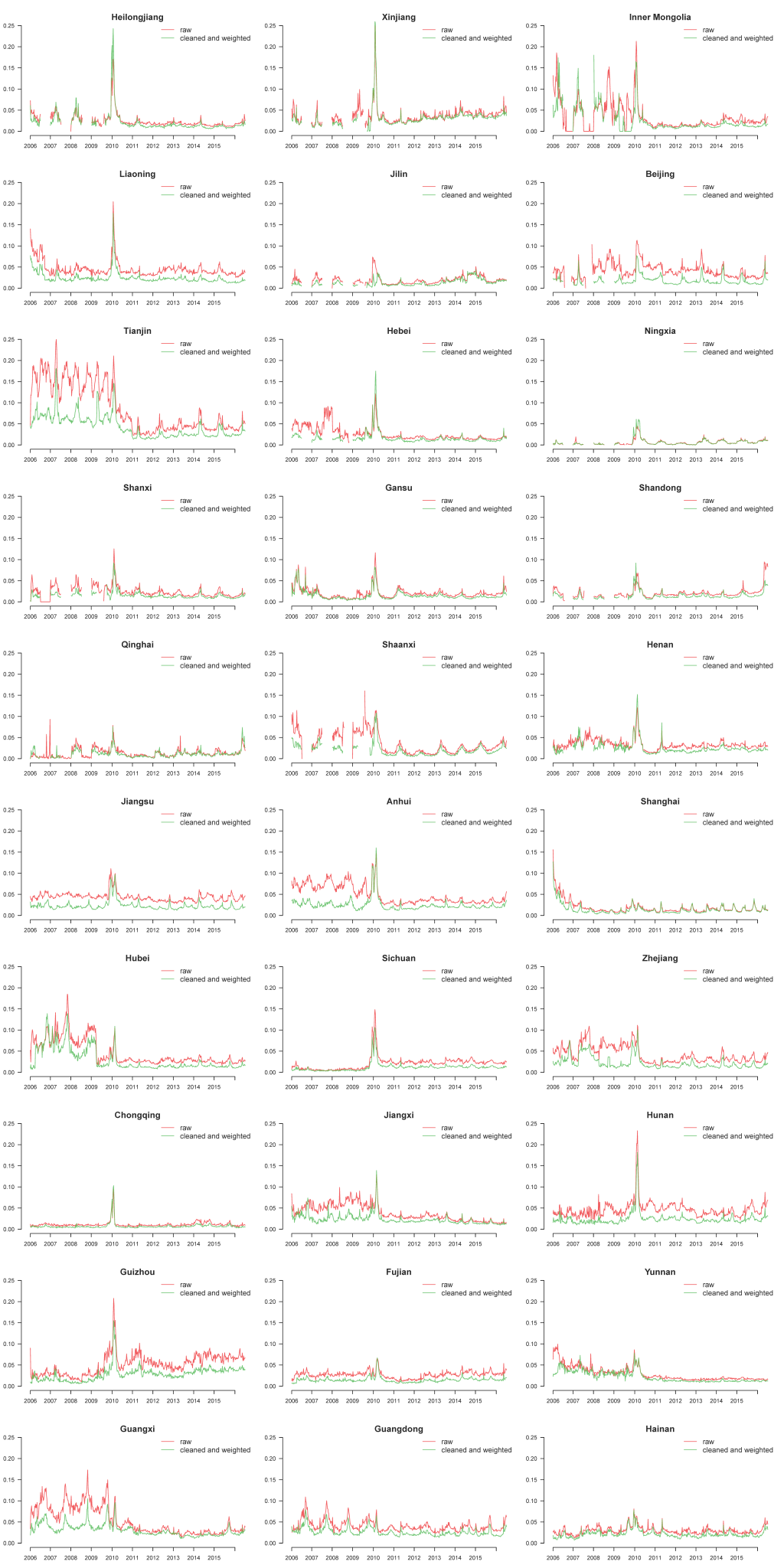


Figure S2. Influenza positive rate before and after data cleaning and weighting, 2006 to 2015.


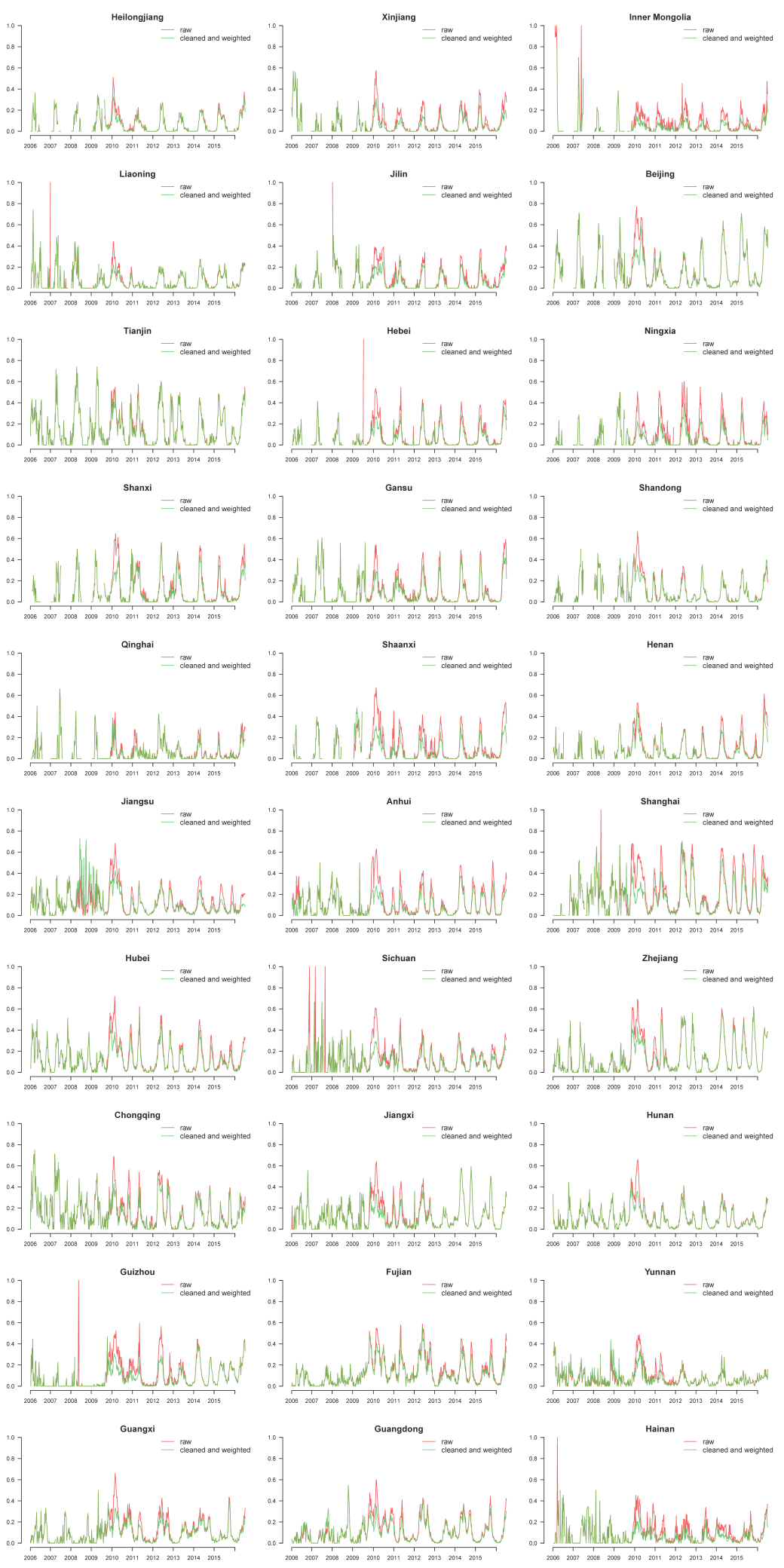


Figure S3. ILI consultation rates in 30 provinces in China, 2006-2015. The provinces were ordered in descending latitude

Figure S4. Laboratory confirmed influenza positive rate in 30 provinces in China, 2006-2015. The provinces were ordered in descending latitude

Figure S5. Influenza-associated ILI outpatient consultations in 30 provinces in China, comparing observed and fitted data, 2006 – 2015 (per 1,000 populations).

Figure S6. Proportion of influenza-associated burden by influenza type/subtype among total burden across 30 provinces in China, 2006 to 2015. The provinces were ordered in descending latitude. (Type/subtype-specific estimates for were zero in Ningxia in 2015)

Figure S7. Associations between GRP per capita ($)(perGRP), disposable income per capital ($)(perDI), population density, health expense rural ($)(perUHCMS), health expense urban ($)(perRHCMS) and average influenza-associated ILI burden 2006-2015 (exclude 2009) and in 2009.

Figure S8. Cluster analysis on influenza-associated burden, socioeconomic and health expenses in 30 provinces in China, 2006 to 2015.
